# Supplementary material for: Facile Fabrication of Fluorine-Free, Anti-Icing, and Multifunctional Superhydrophobic Surface on Wood Substrates
Source: Polymers (Basel). 2022 May 11;14(10):1953. doi: 10.3390/polym14101953 (PMC9143972; doi:10.3390/polym14101953)
Supplement: Supplementary file 1 [file polymers-14-01953-s001.zip › polymers-1703385-supplementary.pdf]

Supporting information

Facile Fabrication of Fluorine-Free, Anti-Icing, and Multifunctional Superhydrophobic Surface on Wood Substrates

1. Anti-fouling property

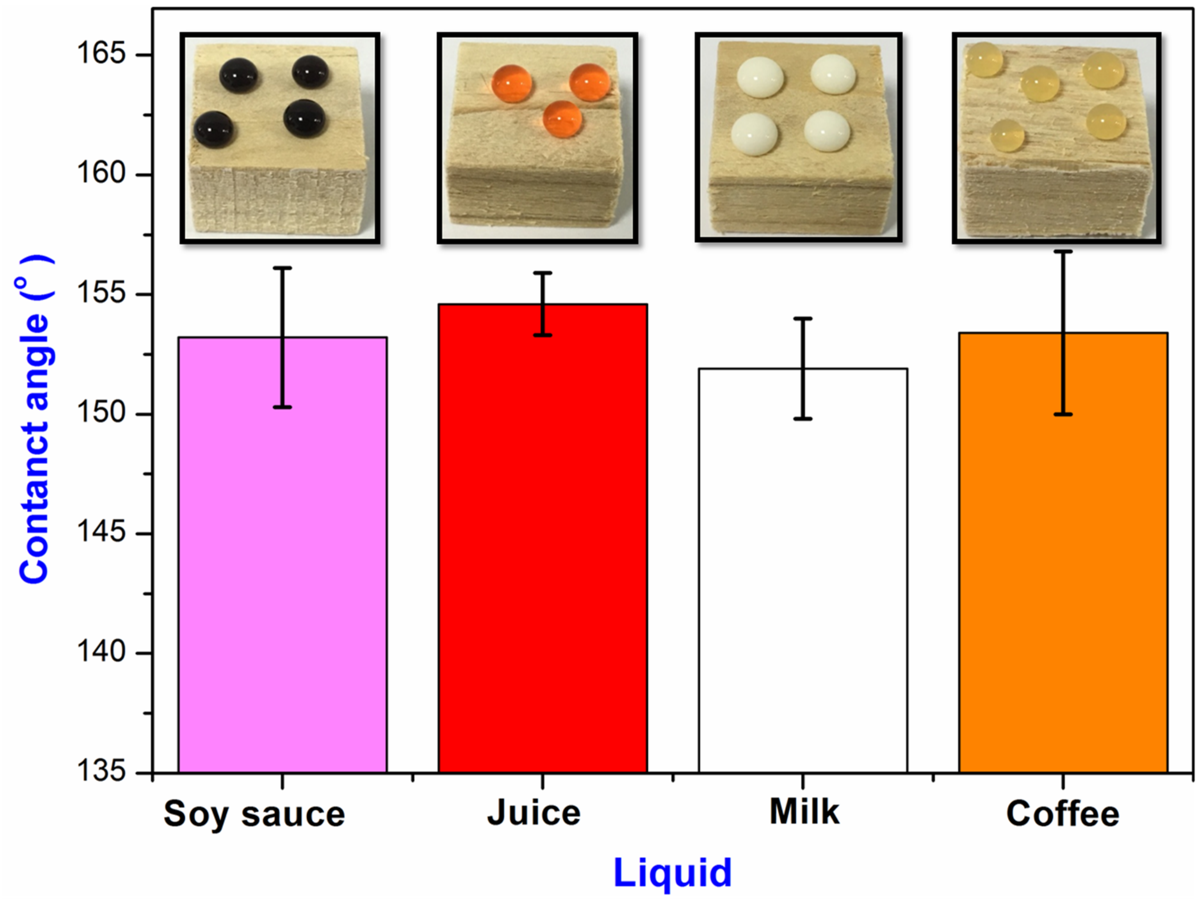

Figure S1. Wettability behavior of SiO_2/PMHOSmodified wood exposed to soy sauce, juice, milk, and coffee.

2. The superhydrophobicity of PMHOS

As shown in Figure S2, the superhydrophobic PMHOS powder was successfully prepared with a WCA of 161°. The water droplets were intended to place on the PMHOS powder surface, but failed. This means that the prepared PMHOS has very low adhesion to water.

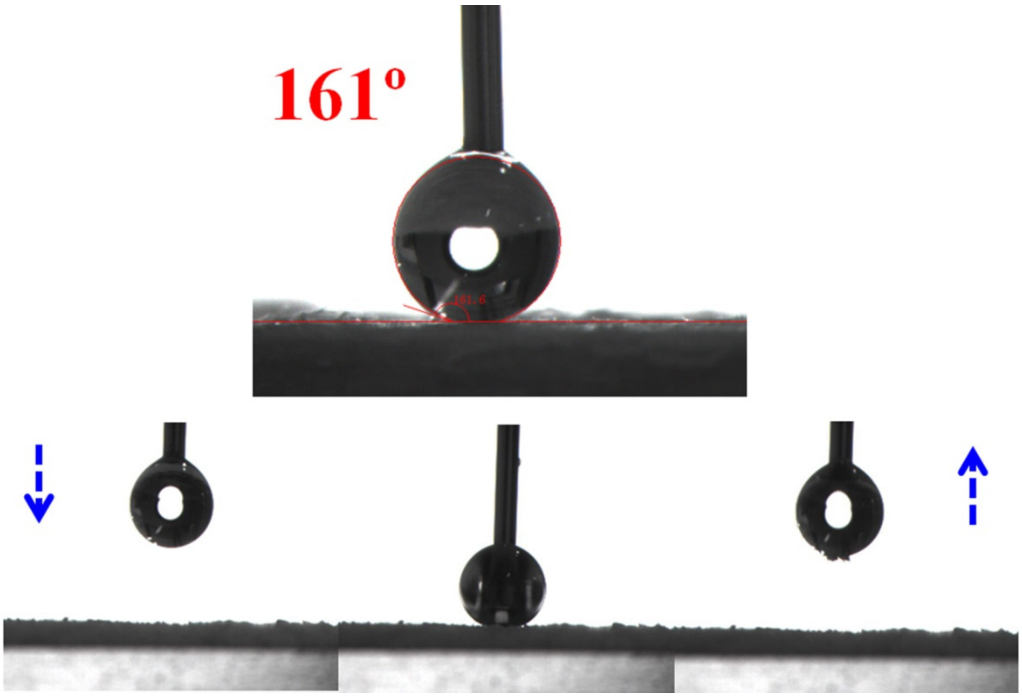

Figure S2. Hydrophobicity test of PMHOS powder.
